# Intravenous Lipid Emulsions in the Prevention and Treatment of Liver Disease in Intestinal Failure

**DOI:** 10.3390/nu13030895

**Published:** 2021-03-10

**Authors:** Fedja A. Rochling

**Affiliations:** Division of Gastroenterology and Hepatology, University of Nebraska Medical Center, Omaha, NE 68198-2000, USA; frochling@unmc.edu; Tel.: +1-402-559-6209

**Keywords:** intestinal failure-associated liver disease (IFALD), parenteral nutrition-associated cholestasis (PNAC), parental nutrition-associated liver disease (PNALD), intravenous lipid emulsions (IVLE), parenteral nutrition (PN), soybean lipid emulsions (SB IVLE), fish oil lipid emulsions (FO IVLE), mixed fish oil-containing lipid emulsions (MFO IVLE), polyunsaturated fatty acid (PUFA), medium-chain triglycerides (MCT), essential fatty acid deficiency (EFAD)

## Abstract

The development of intestinal failure-associated liver disease (IFALD) in pediatric and adult patients on parenteral nutrition is usually multifactorial in nature due to nutritional and non-nutritional causes. The role of lipid therapy as a contributing cause is well-established with the pathophysiological pathways now better understood. The review focuses on risk factors for IFALD development, biological effects of lipids, lipid emulsions and the mechanisms of lipid toxicity observed in laboratory animals followed by a synopsis of clinical studies in pediatric and adult patients. The introduction of fish oil-based lipid emulsions that provide partial or complete lipid replacement therapy has resulted in resolution of IFALD that had been associated with soybean oil-based therapy. Based on case reports and cohort studies in pediatric and adult patients who were at risk or developed overt liver disease, we now have more evidence that an early switch to partial or complete fish oil–based lipid therapy should be implemented in order to successfully halt and reverse IFALD.

## 1. Introduction

The aim of this review is to summarize the current understanding of the etiopathogenesis of intestinal failure associated-liver disease (IFALD) with a special focus on the role of lipids in the reduction or prevention of IFALD in pediatric and adult patients.

Three terms are used to describe liver diseases associated with prolonged use of parenteral nutrition—IFALD, PNAC and PNALD [1].

Intestinal Failure-associated Liver Disease (IFALD) is the preferred term to describe the liver dysfunction encountered in pediatric and adult patients on parenteral nutrition. IFALD reflects more accurately the multifactorial nature of the problem than the preceding terms Home Parental Nutrition (HPN)-related liver disease or HPN-associated liver disease [2]. The risk factors that predispose to liver injury encountered in chronic intestinal failure have been subdivided into nutrient dependent and non-nutrient dependent causes in pediatric and adult individuals. 

Lipid injectable emulsions present a composite of one or more triglyceride-containing oils paired with glycerin and egg phosphatide, which is a phospholipid emulsifier. Lipids provide not only a higher energy content than glucose and amino acids but also provide the fatty acids needed for cell membrane integrity, signal transduction pathways, immune functions, and gene expression regulation to name but a few. 

The micelle or chylomicron-like particle in each emulsion is very similar to the enterally formed chylomicron with regard to size, phospholipid layer and internal triglyceride molecule [3]. The lipid content of each commercially available emulsion ranges from 10–30% with other components such as vitamin E and K, phytosterols and cholesterol being added. The designed particles are also termed pseudo-chylomicrons that, when presented to the cells, release the fatty acid content for β-oxidation. The dosing of the lipid provided in parenteral nutrition depends on factors such as underlying illness, energy expenditure, body weight and the ability to metabolize lipids. In adults, the dosing of IVLE should not exceed 2.5 grams of lipid per kg per day with a lower dosage applying in different scenarios [3]. 

The early diagnosis and recognition of IFALD is important in order to prevent the undesirable outcome of end-stage liver disease with its associated morbidity and mortality and need to organ replacement therapy. It is noted that the early diagnosis of IFALD can present a challenge.

Great progress in the understanding of the pathophysiological processes has been made in recent years, mainly derived from studies in the pediatric patient population, which has resulted in newer lipid formulation and adaptation in care processes. Soybean-based lipid formulations present just one cause of liver injury in patients on parenteral nutrition. 

The lessons learned from the pediatric research have started to benefit the adult patient dependent on parenteral nutrition.

## 2. Definition and Classification of Intestinal Failure (IF) and Intestinal Failure-Associated Liver Disease (IFALD)—Terminology

The original definition of intestinal failure by Fleming and Remington from 1981 has evolved to a consensus statement that has been endorsed by ESPEN [4,5]. Intestinal failure (IF) is defined as the “reduction of gut function below the minimum necessary for the absorption of macronutrients and/or water and electrolytes, such that intravenous supplementation (IVS) is required to maintain health and/or growth” [5]. 

The term Intestinal Failure-associated Liver Disease (IFALD) has been preferred over the older term parenteral nutrition-associated liver disease (PNALD) because liver disease in pediatric and adult patients is, in most instances, due to multiple contributors [6]. 

Khalaf and Solkol recently defined IFALD as a spectrum of liver disease in patients with intestinal failure that includes cholestasis with or without progression to cirrhosis, steatohepatitis and gallbladder disease in the setting of prolonged PN use and the absence of other obvious causes [1]. The same authors provided us also with definitions for Parenteral Nutrition-Associated Cholestasis (PNAC) and Parental Nutrition-Associated Liver Disease (PNALD.) They defined PNAC as a serum-conjugated bilirubin >1–2mg/dL and more than 20% of total bilirubin in patients who have been on PN for longer than 2 weeks in the absence of other causes of cholestasis [1]. PNALD was defined as a spectrum of liver disease that included cholestasis progressing to cirrhosis, steatohepatitis and gallbladder disease in patients on PN where other causes of liver disease have been excluded [1].

Liver dysfunction is usually detected by increased liver blood tests such as bilirubin, alkaline phosphatase or transaminases. Further, the degree or severity of liver dysfunction can be categorized based on radiological features as seen on sonography, CT or MRI imaging of the liver, elastography, and the gold standard, liver biopsy.

## 3. Risk Factors, Epidemiology and Natural History of IFALD

Since the original association of soybean-based lipid emulsions with liver injury, more investigators have reported specific risk factor associations with the development of IFALD in both, pediatric and adult, patient cohorts [7]. 

The reported incidence of IFALD is between 40–60% of pediatric patients and between 15–40% of adult patients. More specifically, recent data from the North American based Pediatric Intestinal Failure Consortium (PFIC) would indicate that some 38% of patients had conjugated bilirubin levels >4 mg/dL at baseline with a corresponding 4xfold increased mortality risk when seen prior to the advent of fish-oil based lipid emulsion. Some 28% of patients seen by the PFIC group did not have IFALD at time of presentation [8].

In their seminal work, Messing et al. in France followed a cohort of adult patients in France between 1986 and 1996 and reported a prevalence of complicated home parenteral nutrition-related liver disease of 26%+/−9% at 2 years and 50%+/−13% at 6 years with liver disease accounting for 22% of all deaths [9].

The identified risk factors for the development of IFALD in infants, children and adults differ and also overlap to a certain extent. This review focuses on the role of lipid emulsion in IFALD management. It should be acknowledged that clinically it is usually a combination of risk factors that seem to drive the development and evolution of IFALD as noted in Table 1.

Table 1 Patient-dependent and treatment-related contributors and risk factors to liver disease in adult and pediatric patients [2].

## 4. Biological Effects of Lipid

Complex lipids are esters of fatty acids that are made up of an alcohol component and one or more fatty acid molecules. When fatty acids are not esterified, they are named free fatty acids. Fatty acids contain an even number of carbon atoms. The fatty acids are called saturated, if they do not contain double bonds and unsaturated, if they contain one or more double bonds [10]. Table 2 and Table 3 show the commonly encountered saturated and unsaturated fatty acids.

Lipid emulsions as part of PN provide non-protein energy and allow for reduced amounts of carbohydrate provision, thus lowering the potential adverse impact of high-carbohydrate intake associated lipogenesis. 

In addition, lipids provide the essential fatty acids ω-6 polyunsaturated fatty acid (PUFA) linoleic acid (LA) and the ω-3 PUFA α-linolenic acid (ALA), both of which are plant synthesized. 

Essential fatty acids are named essential because they cannot be synthesized de novo and have therefore to be provided exogenously via enteral or parenteral routes. The essential fatty acids will eventually be utilized to build prostaglandins, thromboxanes, leukotrienes and lipoxins [10].

Lipids also allow for the provision of building blocks for cell membrane and fat-soluble vitamins [11]. Linoleic acid will undergo conversion to arachidonic acid while ALA is the precursor of eicosapentanoic acid (EPA) and docosahexanoic acid (DHA) [12]. 

Lipids used in PN are made up of triglycerides which are classified as (i) medium-chain triglycerides with two or three fatty acids and an aliphatic tail of 6–12 carbon atoms, (ii) long-chain fatty acids with 14–20 carbon atoms and (iii) very long chain fatty acids with more than 22 carbons.

The intravenous administration of lipid emulsions implies that there is no enteral digestion involving chylomicron formation, bile acid aided emulsification or pancreatic enzyme driven hydrolysis. Thus, IVLEs need to be prepared in such a way that they can enter easily into a hydrophilic environment. 

**Table 2:** Saturated Fatty Acids [10] with modifications.

Common Name	Number of Carbon Atoms	Occurrence
Acetic	2	Major end product of carbohydrate metabolism by rumen organisms
Butyric	4	In certain fats in small amounts (e.g., butter) End product of carbohydrate fermentation by rumen organisms—cecum of herbivores; human colons
Valeric	5	Minor product of gut microbiome [13] May have a role in inhibition of Clostridioides difficile infection [14]
Caproic	6	
Lauric	12	Spermaceti, cinnamon, palm kernel, coconut oils, laurels, butter
Myristic	14	Nutmeg, palm kernel, coconut oils, myrtles, butter
Palmitic	16	Animal and plant fats
Stearic	18	

**Table 3:** Unsaturated Fatty Acids [10].

Number of Carbon Atoms Number and Positions of Common Double Bonds	Family	Common Name	Systematic Name	Occurrence
Monoenoic Acids (one double bond)				
16:1;9	ω 7	Palmitoleic	Cis-9-Hexadecenoic	In nearly all fats
18:1;9	ω 9	Oleic	Cis-9-Octadecenoic	Possibly most common fatty acid in natural fats; particularly high in olive oil
18:1;9	ω 9	Elaidic	Trans-9-Octadecenoic	Hydrogenated and ruminant fats
Dienoic Acids (two double bond)				
18:2; 9,12	ω 6	Linoleic	all-cis—9,12 -Octadecadienoic	Corn, peanut, cottonseed, soybean, many plant oils
Trienoic Acids (three double bonds)				
18:3; 6,9,12	ω 6	γ-Linolenic	all-cis—6,9,12 -Octadecatrienoic	Some plants, e.g., oil of evening primrose, borage oil. Minor fatty acids in animals
18:3; 9,12,15	ω 3	α-Linolenic	all-cis—9,12,15 -Octadecatrienoic	Frequently found with Linoleic acid but particularly in Linseed oil
Tetraenoic Acids (four double bonds)				
20:4; 5,8,11,14	ω 6	Arachidonic	all-cis -5,8,11,14- Eicosatetranoic	Found in animal fats. Important component of phospholipids in animals
Pentaenoic Acids (five double bonds)				
20:5; 5,8,11,14,17	ω 3	Timnodonic	all-cis -5,8,11,14,17- Eicosapentanoic	Important component of fish oils, e.g., cod, mackerel, menhaden, salmon oils
Hexaenoic Acids (six double bonds)				
20:6; 4,7,10,13,16,19	ω 3	Cervonic	all-cis-4,7,10,13,16,19- Docosahexanoic	Fish oils, algal oils, phospholipids in brain

## 5. Lipid Emulsions

The introduction of the first commercial fat emulsion by Arvid Wretlind in 1961 was based on a soybean oil-based lipid mixed with egg yolk [15,16]. Intralipid turned out to have a very good safety profile based on data collected in Sweden between 1965–1978 with only eight reports of suspected adverse reactions for a total of 16 million units used [15]. 

The progress made by 1974 allowed for administration of customized “all-in-one” solutions, the so-called “3-in-1” PN solutions with amino acids, dextrose and customized electrolytes [17]. The observation that soybean-based emulsions had a negative impact on cellular immune function prompted the development of initially two oil and later three and four oil-based lipid emulsions [11,18,19]. 

The aim of all lipid emulsions is to provide a suspension of oil in an aqueous medium that mimics chylomicrons. Inherently, these emulsions are unstable and tend to undergo aggregation, creaming and coalescence into larger droplets. Egg yolk phospholipids, sterile water for injection, glycerol as well as sodium hydroxide for pH stabilization provide the major emulsion agents to complement with the various oils being used [3].

Lipids are composed of fatty acid monomers that are defined as short chain fatty acids when they have 2–4 carbons, medium chain fatty acid with 6–12 carbons, long chain fatty acids with 14–21 carbons and very long chain fatty acids when more than 22 carbons are present.

Table 4 shows the currently available Intravenous Lipid Emulsions (IVLE) in the United States and the European Union based on period of availability and lipid source.

## 6. Mechanisms of Lipid Toxicity observed in Laboratory Animals and Clinical Studies in Pediatric and Adult Patients

The currently available IVLEs fall into five categories of oils being derived from soybean, safflower, coconut, olive or fish oils. Each of these IVLEs has distinct pro-inflammatory or less inflammatory profiles that result in specific benefits and side effect profile.

Soybean oil based IVLE has a high concentration of PUFA with a ratio of LA to ALA of 7:1 and consists of 25% of the non-essential omega-9 fatty acid oleic acid. Soybean oil is plant-derived, which makes it naturally high in phytosterols. Typically, phytosterol such as stigmasterol, campesterol and sitosterol, are not absorbed in the gastrointestinal tract. If these compounds are given intravenously, they tend to accumulate in the hepatocytes resulting in inhibition of 7-α hydroxylase, which, being a rate limiting enzyme in the bile acids synthesis pathway, would result in a reduction in bile acid synthesis and bile flow. The clinical picture of cholestasis would be expected [21,22,23]. One mechanism that has been described, based on studies in HepG2 cells, involves stigmasterol acetate mediated suppression of ligand-activated expression of FXR target genes [24]. In addition, phytosterols integration into the membranes of red blood cells results in accelerated hemolysis [22]. The increased accumulation of plasma and RBC membrane phytosterols in newborns, who are on TPN, appears to correlate significantly with the amount and duration of PN infused [25].

Safflower oil based IVLE became available in 1980 and has, compared to a soybean oil, a higher concentration of linoleic acid and a lower concentration of α-linolenic acid (ALA.) An association of numbness, paresthesia, weakness, inability to walk, painful legs and visual blurring in a 6-year old girl, who had been on 5 months of high linoleic acid and low linolenic acid IVLE, has been described with resolution of symptoms upon correction of linolenic acid deficiency [26].

Coconut oil is the source of medium-chain triglycerides (MCT) in IVLE, usually in the form of 6-12 carbon long capric and caprylic acid. MCTs do not accumulate in the liver, are protein sparing and resistant to peroxidation. The disadvantage of the sole use of MCTs is that they are devoid of any essential fatty acid content and thus predispose the patient to Essential Fatty Acid Deficiency (EFAD) if used as the sole source of lipids. MCTs therefore have to be combined with oils that are higher in content of essential fatty acids [27].

Along the same lines, olive oil contains less than 1% ALA and 10% LA and therefore also needs a blending with soybean-based oil to prevent development of essential fatty acid deficiency states. IVLEs that include olive oil - based monounsaturated fatty acids such as oleic acid, are less pro-inflammatory then soybean-based therapies [27].

Fish oil contains omega-3 fatty acids and higher levels of alpha-tocopherol, a potent antioxidant.

The etiology of fatty acid induced liver injury is felt to be due to a pro-inflammatory environment. The main culprit in soybean based IVLE is felt to be the pro-inflammatory omega 6 fatty acids. This is even more so the case when soybean based IVLE is administered at doses greater than 1 g/kg body weight. Soybean based IVLE is a rich provider of omega 6 fatty acids that are the precursors of arachidonic acid, which in turn, through the cyclooxygenase and lipoxygenase pathways, result in the production of pro-inflammatory process prostanoids and leukotriences.

On the other hand, the beneficial effects of omega-3 fatty acids are attributed to their anti-inflammatory properties. The receptor GPR 120 is located on Kupffer cells and is a key mediator of Omegaven (an omega 3 fatty acid product) activity, with a distinct anti-inflammatory attribute [10,28].

Gura et al. were the first to show reversal of cholestasis in infants who had developed IFALD on soy oil based IVLE by giving fish oil based Omegaven [29]. 

In a follow up, Gura et al. compared safety and efficacy outcomes of a fish-oil-based fat emulsion in 18 infants with short-bowel syndrome who had developed cholestasis (serum direct bilirubin level of > 2mg/dL) while receiving soybean emulsions with those from a historical cohort of 21 infants with short-bowel syndrome who also developed cholestasis while receiving soybean emulsions. The primary end point was time to reversal of cholestasis as defined by three consecutive measurements of serum direct bilirubin level of < or = 2 mg/dL. Gura et al. showed that among survivors, the median time to reversal of cholestasis was 9.4 and 44.1 weeks in the fish oil and historical cohorts, respectively. Subjects who received fish-oil-based emulsion experienced reversal of cholestasis 4.8 times faster than those who received soybean emulsions and 6.8 times faster in analysis adjusted for baseline bilirubin concentration, gestational age, and the diagnosis of necrotizing enterocolitis. A total of two deaths and no liver transplantations were recorded in the fish-oil cohort and seven deaths and two transplantations in the historical cohort. The provision of fish oil-based fat emulsion was not associated with essential fatty acid deficiency, hypertriglyceridemia, coagulopathy, infections, or growth delay. Gura et al. concluded that parenteral fish oil-based fat emulsions are safe and effective in the treatment of parenteral nutrition-associated liver disease [30].

These observations have since then been repeated in multiple other centers. Calkins et al. had previously observed that 75% of children with IFALD who receive a six-month course of IV fish oil (FO) experienced cholestasis reversal in comparison to 6% of children who received soybean oil [31]. Calkins et al. subsequently aimed to quantify changes in polyunsaturated fatty acids, phytosterols, cytokines, and bile acids in children who received FO for IFALD treatment [32]. They replaced soy-based Intralipid with fish-oil based Omegaven and treated 21 patients with fish oil of which 14 completed the study. Calkins et al. reported that, at study initiation, the median patient age was 3 months (interquartile range [IQR], 3–17 months), and mean ± standard deviation direct bilirubin was 5.6 ± 0.7 mg/dL (*n* = 14). Cholestasis reversed in 79% of subjects. Eicosapentaenoic and docosahexaenoic acid was greater than baseline (*p* < 0.001, all time points.) Linoleic and arachidonic acid and sitosterol and stigmasterol were less than baseline (*p* < 0.05, all time points). Three- and 6-month interleukin-8 (IL-8) and total and conjugated bile acids were less than baseline (*p* < 0.05.) Baseline IL-8 was correlated with baseline direct bilirubin (*r* = 0.71, *p* < 0.01). Early changes in stigmasterol and IL-8 were correlated with later direct bilirubin changes (*r* = 0.68 and 0.75, *p* < 0.05) [32]. Thus, intravenous fish oil treatment led to reversal of cholestasis. A reduction in plasma phytosterols, cytokines, and bile acids and a change in the erythrocyte content of polyunsaturated fatty acids were shown. Further, the level of IL-8 correlated with direct hyperbilirubinemia, and early changes in stigmasterol and IL-8 correlated with later changes in direct bilirubin, leading the authors to assume that these biomarkers may play a role in IFALD and be indicators of treatment response [32].

Puder et al. characterized the fatty acid profiles in infants with intestinal failure-associated liver disease. They studied two cohorts prospectively and noted no differences in demographics or anthropometrics between patients who received standard SO (SO-S) (*n* = 14, range of dose 2.06–3.31 g/kg/day) and reduced SO (SO-R) (*n* = 35, range of dose 0.90–1.34 g/kg/day.) The markers for EFAD (Triene: tetraene ratios) were higher in patients who received SO-R (*p* = 0.0009) and no patients developed biochemical EFAD. Therefore, soybean oil-based lipid emulsions appear to provide adequate essential fatty acids at a dose of 1 g/kg/day [33].

Pastor- Clerigues et al. measured the inflammatory and profibrotic markers in serum of ten adults with long term PNALD and in culture supernatants of monocytes [34]. This was a descriptive study that looked at different soybean and fish parenteral lipid emulsions in adults who developed PNALD secondary to long-term PN. They showed in two adult patients with soybean-based IVLE induced IFALD, that 100% fish oil Omegaven^®^ lipid treatment restored the hepatic enzymes, fibrotic and inflammatory cytokine markers to normal values. In addition, fish-oil based treatment showed strong anti-inflammatory properties related with a broad spectrum of innate and adaptive T_H_1 and T_H_2 responses as well as an inhibitory effect on profibrotic TGFβ1 and MMP-9 secretion [34]. The authors showed that reintroduction of a mixture of ω-6/ω-3/ω-9/MCT fatty acid emulsion SMOFlipid^®^ increased the liver enzymes, inflammatory and pro-fibrotic markers in serum to similar levels to those observed before starting Omegaven infusion. As the authors noted, since Omegaven does not provide the required essential fatty acid load for optimal nutrition, the ideal situation for long-term parenteral nutrition-associated liver disease in adults would be to alternate 2–4 months of parenteral nutrition with Omegaven^®^ lipids with 2 months of an equilibrate fish oil SMOFlipid^®^ in repetitive cycles [34].

The relative roles of phytosterols and α-tocopherol in IFALD have been studied. Soybean oil is rich in phytosterols, poor in α-tocopherol, while fish oil contains more α-tocopherol and less phytosterols. Prior studies have shown that medium-chain triglycerides (MCTs) and α-tocopherol have anti-inflammatory properties. 

Baker et al. in 2019 asked whether medium-chain triglycerides (MCTs) or α-tocopherol contribute additional systemic anti-inflammatory properties to fish oil-based IVLEs in a murine model of PN-induced liver injury. In a series of two experiments the group aimed to (1) determine the optimal FO: MCT ratio to maximize the anti-inflammatory benefit of the IVLE while preventing hepatic steatosis and biochemical EFAD and (2) to determine the effect of additional α-tocopherol in FO- and FO/MCT-IVLE [35]. The study showed that MCT and α-tocopherol can result in a downregulation of the systemic inflammatory response in a murine model of PN-induced liver injury. Mice that received a combination of FO/MCT-ILE showed an attenuated systemic inflammatory response to endotoxin challenge, as measured by serum IL-6 and TNF-α, compared with mice that received an ILE with FO alone [35].

Fell et al. in 2019 showed in a murine model that α-tocopherol added to soybean oil was able to preserve normal hepatic architecture and normal expression of regulators of hepatic fat metabolism (Acetyl CoA Carboxylase 2 and Peroxisome Proliferator Activated Receptor-gamma) in a murine model of PN-induced liver injury in which mice are enterally fed a PN solution. Fell et al. showed that an IVLE formulated in the laboratory using soybean oil that did not contain added α-tocopherol was not able to protect from PN-induced hepatic steatosis and dysregulation of hepatic fat metabolism [36].

Bechynska et al. in an effort to understand the underlying mechanism of PNALD took a different approach and determined serum and liver lipidome, liver proteome, liver bile acid profile as well as markers of inflammation oxidative stress in rats administered either ω-6 PUFA based lipid emulsion (Intralipid) or ω-6/ω-3 PUFA blend (Intralipid/Omegaven) via the enteral or parenteral route in rats [37]. They showed that PN administered omega-3 polyunsaturated fatty acids (ω-3 PUFA), compared with parenterally administered Intralipid, and was associated with 1. Increased content of eicosapentaenoic (EPA) - and docosahexaenoic (DHA) acids-containing lipid species; 2. Higher abundance of CYP4A isoenzymes capable of bioactive lipid synthesis and the increased content of their potential products (oxidized EPA and DHA); 3. Downregulation of enzymes involved CYP-450 drug metabolism what may represent an adaptive mechanism counteracting the potential negative effects (enhanced ROS production) of PUFA metabolism; 4. Normalized anti-oxidative capacity and 5. Physiological BAs spectrum. All these findings may contribute to the explanation of ω-3 PUFA protective effects in the context of PN [37].

The role of the GI tract in modulating PN induced liver injury remains unclear. It is well-known, however, that gut mucosal atrophy occurs in animals and patients who are fed exclusively with PN [38]. Parenteral nutrition leads to mucosal atrophy due to decreased intestinal epithelial cell proliferation and increased apoptosis, loss in mucosal epithelial barrier function, decline in the size of the crypt/villus complex, and reduction in intestinal length [39,40,41]. 

Freeman et al. had reported in 2015 that small intestinal architecture and barrier function are significantly altered without effective TLR4 signaling in a TPN mouse model [39]. 

Considering the potential role of luminal nutrient deprivation in PN-induced liver disease, Guzman et al. in 2020 hypothesized that it is this state of luminal content deprivation during TPN therapy that induces alterations in gut-derived signaling, contributing to liver and gut injury [42]. Guzman et al. used one-week-old farm raised neonatal piglets and divided them into a TPN-fed cohort that was deprived of any luminal feeds and an enterally fed group. Guzman et al. noted significant elevation of cholesterol-7-alpha-hydrolase and bile in TPN fed piglets. Cholesterol 7 alpha-hydroxylase is a rate-limiting step in bile acid synthesis. They showed a reduced expression of the gut FXR mRNA expression in animals on TPN as compared to those on enteral nutrition [42]. In addition, Guzman et al. showed a reduction in the thickness of muscularis mucosa as part of the gut mass differences with TPN [42].

In 2020, Calkins et al. followed up on the previous observations on the role of microRNA-122 as predictive biomarker of cholestasis, steatosis and fibrosis in animals and humans [43]. Circulating microRNA-122 has been identified as a biomarker for drug-, viral-, alcohol- and chemical-induced liver injury [44,45]. Calkins et al. measured miRNA-122 in a prospective cohort of children with IFALD who received fish oil and whose IFALD resolved with fish oil [43]. Calkins et al. showed that plasma miR-122 decreases with fish oil treatment in a cohort of 14 children with IFALD correlating with a fall in conjugated bilirubin. In addition, they also observed after treatment with fish oil a downregulation of reactive oxygen species, heme metabolism, coagulation, adipogenesis, IL-6-Janus kinase-signal transducer and activator of transcription (JAK-STAT) 3, IL-2-STAT5, transforming growth factor-beta, TNF-alpha, inflammatory response, mammalian target of rapamycin gene families and miR-122 target genes [43].

## 7. Management Approaches

The approach to management of IFALD in pediatric and adult patients has to be proactive taking into account the specific risk factors. The prevention and treatment of established IFALD calls for an integrated team-based approach towards these patients with members of the team being dieticians, laboratory staff, pharmacists and physicians. Monitoring includes lab testing, sonography integrated with elastography and in some select instances, when the diagnosis is in question, the performance of a liver biopsy. This approach naturally may differ between neonatology, pediatric and adult practices depending on local expertise. The approach to these patients has to be based on the foundation of constant awareness of the at-risk profile of the pediatric and adult patient although. The next paragraphs will focus on some key aspects of the management approaches in pediatric and adult patients that can be distinguished according to reversal or prevention of IFALD. Most of the literature has focused on observations in the pediatric study cohorts [46].

### 7.1. Reversal of Cholestasis/IFALD in Pediatric Patient Population

Since 2000, a number of retrospective and prospective publications have been published that have addressed methods to reverse cholestasis. These approaches have focused on discontinuation or reduction in soybean IVLE [47,48], substitution of fish oil-based emulsion while receiving soybean emulsions [30,31,32,49,50,51,52,53,54], or substitution with a SMOF (soybean, coconut, olive, and fish oils) or Lipofundin IVLE [55,56]. 

The simple, temporary discontinuation of soybean-based IVLE in infants, while it may result in normalization of bilirubin, is associated with growth retardation and EFAD and therefore, does not present a viable, long-term solution. 

The seminal observation by Gura et al., reported initially in case report format, involved the substitution of soybean-based IVLE with fish oil-based IVLE and resulted in normalization of bilirubin. The benefits of fish-oil based IVLE have since then been shown in multiple studies since [30,31,32,49,50,51,52,53,54]. The major drawback of pure fish oil therapy remains the development of EFAD. Concerns remain also because of development of Burr cell anemia from hemolysis in pediatric patients on prolonged fish oil based- therapy and abnormal platelet function resulting in increased bleeding risk [57,58,59].

The question remains what to do with patients who improve after fish-oil based therapy. Wang et al. in 2019 reported their follow up experience in a prospective observational study of children with soybean oil induced IFALD [54]. Wang et al. showed that fish oil effectively treated cholestasis. Once the IFALD had resolved, Wang et al. allowed for resumption of soybean based IVLE in 27 patients which was associated with a cumulative cholestasis redevelopment in 26%. This raises the question whether long-term fish oil based IVLE may be warranted to prevent end-stage liver disease in pediatric patients [54]

The long-term use of fish oil-based IVLE has been addressed in the 2018 ESPGHAN/ESPEN/ESPR/CSPEN guidelines [60]. The guidelines state that the use of pure fish oil-based IVLE is not recommended for general use in pediatric patients. A short-term rescue effort less than 15 days may be considered in patients with progression to severe IFALD based on case report data [61,62]. Further, a Consensus Statement from the International Summit “Lipids in Parenteral Nutrition” held in Miami FL in November 2018, stated that in cholestatic (IFALD) pediatric patients on chronic PN, pure fish oil-based IVLE can be used as rescue therapy in severe IFALD (bilirubin >2 mg/dL) [63]. Pure fish oil-based ICLE should not be used over a prolonged period of time [63]. The use of a composite IVLE that includes fish oil as first line treatment for IFALD is recommended [63]. 

### 7.2. Prevention of Cholestasis/IFALD in Pediatric Patient Population

The at-risk profile of pediatric patients for the development of IFALD has been discussed above. The recognition of the at-risk pediatric patient should prompt the use of a composite IVLE mixture. This approach is based on randomized controlled trials and a prospective cohort study. 

Goulet et al. in 2010 conducted a RCT of 28 patients who were given SMOF lipid 20% or standard soybean based IVLE. SMOF 20% was made up of 30% soybean oil, 30% medium-chain triglycerides, 25% olive oil, and 15% fish oil plus α-tocopherol [64]. Goulet et al. showed that total bilirubin decreased significantly in the SMOF lipid 20% group during the 4-week infusion period, whereas it increased in the soybean oil group [64]. SMOF lipid 20% significantly increased the long-chain ω-3 FAs EPA and DHA as well as the ratio of ω-3:ω-6 FAs which is expected to result in improved visual and cognitive function because of the structural role of ω-3 DHA in brain and brain neuronal membranes [64].

Diamond et al. compared SMOF lipid to Intralipid in a multicenter blinded randomized controlled trial of 24 infants studied between June 2020 and September 2011 in Canada [65]. Diamond et al. showed that patients who received SMOF lipid had a lower conjugated bilirubin than those who received Intralipid.

Lam et al. in 2018 report on a cohort study conducted between 2000 and 2016 in Toronto, Canada. SMOFlipid had become available in 2013 in their center. Lam et al. showed that prolonged use of SMOFlipid in hospitalized children was associated with significantly lower conjugated bilirubin compared with Intralipid. This suggested that SMOFlipid could be used preferentially over Intralipid in hospitalized children with varied medical conditions (such as those with inflammatory bowel disease, intussusception, and bowel obstruction), including those outside the neonatal/infantile period [66].

In 2020, Casson et al. reported a retrospective study that compared the infant patients from two intestinal rehabilitation centers in Dallas, Texas and Edmonton, Alberta [67]. The different strategies at initiation of PN at age 7–8 days compared the incidence and degree of cholestasis between infants with intestinal failure receiving either SMOF or Intralipid. The hypothesis was that infants who are initiated and maintained on SMOF will have a reduced incidence and severity of cholestasis compared to those initially receiving Intralipid [67]. The authors reported that SMOFlipid use in infants did not significantly decrease the incidence or degree of cholestasis. The use of SMOFlipid however was associated with a faster resolution of hyperbilirubinemia once it had developed on Intralipid. SMOFlipid may be most beneficial for infants tolerating no enteral nutrition [67].

### 7.3. Prevention of IFALD in the Adult Patient Population

In patients who require home PN for longer than 6 months, soybean-based IVLE should not exceed 1 grm/kg/day to prevent liver complications [68]. In addition, prevention measures included timely management and prevention of sepsis, strict avoidance of hepatotoxic medication and alcohol consumption, maintenance of enteral intake and cyclical administration of PN [68]. In addition, the rate of infusion of if IVLE should not exceed 0.11 g/kg/h in order to avoid fat overload syndrome [69]. There are presently no randomized controlled data available that establish superiority of one IVLE over another in the primary prevention of IFALD.

### 7.4. Reversal of IFALD in Adult Patient Population

Most of the interventions used to reverse established IFALD in adult is derived from the more robust literature reported in pediatric patients. The predominantly cholestatic IFALD seen in children differs from the mainly steatotic picture seen in adults, who may have preceding liver disease that antedates IFALD such as alcoholic liver disease, NAFLD or viral hepatitis. 

Reversal of IFALD in adults using different IVLE is therefore based on case reports and case series. 

Pironi et al. in 2010 reported 8 month treatment with fish oil based IVLE of a 58 year old patient with IFALD resulting in improvement of steatosis and inflammation, but no change in fibrosis on serial liver biopsies [70]. 

Burns et al. in 2013 used Omegaven in an adult patient with IFALD [71]. Jurewitsch et al. converted a 75-year old woman with cholestatic IFALD from 20% Intralipid to Omegaven with biopsy proven resolution of cholestasis [72]. 

Moyes et al. in 2012 reported the devlopment of jaundice in a 43-year old man who had received 6 months of a mixture of refined olive oil (80%) and soybean oil (20%) with the liver biopsy showing severe acute cholestasis, minimal steatosis without inflammation and portal fibrosis all consistent with PNALD [73]. Subsequently the patient developed portal hypertension which resulted in a gastric variceal bleed. A change to SMOFlipid 20% was implemented which provided the patient with a mixture of soybean (30%), medium-chain triglycerides (30%), olive oil (25%) and fish oils (15%) [73]. Over 16 months of treatment with SMOFlipid20% the patient developed biochemical improvement, resolution of portal hypertension and a decrease in liver stiffness assessed by serial fibroscan [73]. The authors attributed the improvement to the ω-3 fatty acids in fish oil that result in prevention of hepatic injury by inhibition of de novo lipogenesis and stimulation of fatty acid β-oxidation, thus reversing hepatic steatosis [73].

Venecourt-Jackson et al. in 2013 reported the case of a 53-year-old man who had received some 27 months of cyclically administered ClinOleic lipid formulation (20% soybean oil, 80% olive oil) and had developed IFALD (bilirubin 31.29 mg/dL.) The switch to the total fish-oil based emulsion (Omegaven) resulted, within 8 weeks, in normalization of transaminases and a trough bilirubin of 3.68 mg/dL) [74] 

Park et al. in 2020 reported two cases of IFALD in adult patients. Case 1 had been on 11 months of PN with a lipid supply consisting of soybean, olive, medium chain triglycerides (MCT), and fish oils (6:5:5:4 ratio.) Fish-oil based therapy (0.15 grm/kg/d) along with SMOFlipid (0.3–0.6-g/kg/d) resulted in resolution of IFALD after 59 days of therapy. The patient has since been maintained on SMOFlipid at 1 grm/kg/d [75]. Case 2 involved administration of fish-oil treatment after 50 days of receiving PN. Fish oil was given daily at a dose of 5 g/d, and the combination of soybean, olive, medium chain triglycerides (MCT), and fish oils (6:5:5:4 ratio) at 20 g/d was administered 3 days a week (average fish oil, 0.14 g/kg/d) [75]. In both cases Park et al. reported that fish oil at a dose of 0.14–0.24 grm/kg/d was well tolerated during the entire period of administration. It should be noted that fish oil was added, not substituted, to the IVLE in both cases.

These case reports provide evidence that a switch to a partially or completely fish oil –based lipid emulsion therapy in adult patients with IFALD can result in improvement or even normalization of liver function with potential for reversal of advanced fibrosis and resolution of portal hypertension.

## 8. Conclusions

The management of IFALD in pediatric and adult patients calls for a multidisciplinary approach that involves the entire team of care providers. The potentially hepatotoxic role of the first-generation soybean-based IVLE has to be addressed when IFALD has been encountered. 

The non-nutritional management includes the early recognition and treatment of sepsis, management of bacterial overgrowth and recognition that the patient can also have other causes of liver disease. In addition to the standard of care monitoring of liver blood tests, the newer tools of fibrosis assessment, such as fibroelastography, should be engaged in the long-term management of patients that are PN dependent. 

The nutritional management involves the maximization of enteral routes of feeding as this stimulates the enterohepatic circulation. Cyclical administration of PN should be implemented as it allows for a mobilization of fats during non-administration times of PN. 

The role of prophylactic cholecystectomy in long-term PN dependent patients has been reviewed. Prophylactic cholecystectomy (PC) of the non-diseased gallbladder has been recommended in SBS patients when laparotomy is being undertaken for other reasons since some 30% of patients develop cholelithiasis with 2 years of diagnosis of short bowel syndrome [76,77,78]. 

All these are preventative efforts to avoid IFALD development. The management of established IFALD includes an inclusive work up for other causes of liver dysfunction that may still call for a liver biopsy in some instances. Consideration of adding ursodeoxycholic acid, taurine, choline and carnitine has been entertained [68]. 

The nutritional approach should call for the switch from a soybean-based to a pure fish oil based or partially fish oil-based lipid formulation in order to decrease the ω6/ω3 PUFA ratio [68]. Caloric overfeeding with PN needs to be minimized. The evidence for benefit of switching is more robust in pediatric than in adult patients. It is too early to say that the availability of glucagon-like peptide-2 (GLP-2) including the next generation of growth factors such as Glepaglutide will result in improved outcomes in patients with IFALD [79,80].

Finally, while progress has been made in the management of IFALD, transplantation evaluation should still be considered in the increasingly rarer scenario where IFALD does not resolve [81,82].

## Figures and Tables

**Table 1:** Risk Factors for Liver Disease in Adult and Pediatric Patients on Long-term Parenteral Nutrition.

	Risk Factors Adults	Risk Factors Pediatrics
Nutrient Dependent	Energy Overfeeding Glucose Overload Lipid emulsion overload Soybean lipid emulsion >1gm/kg/d Continuous infusion (24/7/365) Contaminants (phytosterols) Antioxidant deficiency Choline deficiency Carnitine deficiency Methionine deficiency Taurine deficiency Essential fatty acid deficiency	High parenteral energy intake Use of soybean-based lipid emulsion (>1g/kg) Dextrose infusion (>7mg/kg/min) Transsulfuration pathway deficiency (choline) Toxicity from plant phytosterols Quality & quantity of amino acids
Non-Nutrient Dependent	Lack of oral feeding Short small bowel remnant Sepsis – CLABSI Small bowel bacterial overgrowth Microbiome Concurrent established hepatotoxic agents such as autoimmune liver disease, viral hepatitis, drugs, alcohol use	Prematurity Low birth body weight Young age at PN commencement Frequent sepsis Long duration of PN Fasting or low enteral intake Presence of SBS with stoma Presence of gastroschisis Presence of intestinal atresia

**Table 4:** Lipid Emulsions for Parenteral Nutrition [19,20].

Generation	Lipid Emulsion	Lipid Source	Commercial Name	Manufacturer	Cost
First Generation 1960s–1970s	Soybean Oil	100% SO	Intralipid^®^ 20%	Fresenius Kabi, Bad Homburg Germany	(a)
Second Generation Since 1985	Soybean Oil Coconut Oil	50% SO 50% CO	Lipofundin^®^ 20%	B. Braun Melsungen Germany	(b)
Second Generation Since 1985	Soybean Oil Coconut Oil	64% SO 36% CO	Structolipid^®^ 20%	TBD	(c)
Third Generation Since 1990	Soybean Oil Olive Oil	20% SO 80% OO	ClinOleic^®^ (ClinoLipid) 20%	Baxter, Deerfield IL USA	(d)
Fourth Generation Since 2000	Fish Oil	100% FO	Omegaven^®^ 10%	Fresenius Kabi, Bad Homburg Germany	(e)
Fourth Generation Since 2000	Coconut Oil Soybean Oil Fish Oil	50% CO 40% SO 10% FO	Lipiderm LipoPlus^®^ 20%	B. Braun Melsungen Germany	(f)
Fourth Generation Since 2000	Soybean Oil Coconut Oil Olive Oil Fish Oil	30% SO 30% CO 25% OO 15% FO	SMOFlipid^®^ 20%	Fresenius Kabi, Bad Homburg Germany	(g)

SO, soybean oil; CO, coconut oil; OO olive oil; FO fish oil; Per Lexicomp cost in USA: 20% (per mL): $0.20 30% (per mL): $0.13. (a) No price info for USA (b) No price info for USA; (c) Per Lexicomp cost in USA: 20%(per mL):$0.20, (d) Per Lexicomp cost in USA: 5 gm/50 mL (per mL): $1.21; 10 g/100 mL (per mL): $0.86, (e) No price info for USA, (f) Per Lexicomp cost in the USA: 20% (per mL): $0.12, (g) No price info for USA.
